# Clinical Profile of PiB-Positive Corticobasal Syndrome

**DOI:** 10.1371/journal.pone.0061025

**Published:** 2013-04-05

**Authors:** James R. Burrell, Michael Hornberger, Victor L. Villemagne, Christopher C. Rowe, John R. Hodges

**Affiliations:** 1 Neuroscience Research Australia, Sydney, New South Wales, Australia; 2 Prince of Wales Clinical School, Sydney, New South Wales, Australia; 3 University of New South Wales, Sydney, New South Wales, Australia; 4 Department of Nuclear Medicine and Centre for PET, Austin Health, Melbourne, Victoria, Australia; 5 Department of Medicine, Austin Health, The University of Melbourne, Melbourne, Victoria, Australia; Georgetown University Medical Center, United States of America

## Abstract

**Background:**

Corticobasal syndrome (CBS) is a multifaceted neurodegenerative disorder characterized by a combination of motor and cognitive deficits. Several different pathological entities, including Alzheimer’s pathology, have been described in association with CBS. The present study aimed to establish clinical, neuropsychological, and neuroimaging features that could be useful in the distinction of CBS due to AD pathology from other CBS cases in life based on [^11^C] Pittsburgh Compound B positron emission tomography (PiB-PET) status.

**Methods:**

Patients with CBS were prospectively recruited from a specialized cognitive disorders clinic. All patients underwent detailed clinical and neuropsychological assessment, with structural imaging using voxel-based analysis of magnetic resonance imaging. Alzheimer’s pathology was detected using PiB-PET imaging, and PiB-positive and PiB-negative groups were compared.

**Results:**

Fourteen CBS patients meeting defined criteria were included (7 male, 7 female; mean age 66.1+/−6.9 years; median symptom duration was 35.5+/−22.6 months) and compared to 20 matched control subjects. Of the 14 patients, 4 were PiB-positive and 10 PiB-negative. There were no significant differences between PiB-positive and PiB-negative CBS patients in age, gender, education, symptom duration, or motor features. PiB-positive patients had greater visuospatial deficits, a higher rate of sentence repetition impairment, and more functional decline. Voxel-based morphometry analyses demonstrated extensive peri-insular and post-central atrophy in both groups, but PiB-positive patients had atrophy that extended to include the posterior part of the left superior temporal gyrus.

**Conclusions:**

Visuospatial function, aspects of language, and the pattern of cerebral atrophy may be useful in distinguishing patients with CBS due to underlying AD pathology.

## Introduction

Corticobasal syndrome (CBS) is characterized by marked clinical and pathological heterogeneity. [1]–[4] While often considered an atypical parkinsonian syndrome, other motor system deficits are common and include asymmetrical limb apraxia – which may be severe and dominate the presentation, dystonia, and myoclonus. [5] In addition, cognitive deficits with progressive disturbance of language and behavior are now regarded as core features of the syndrome. [3], [6]–[8] Pathologically, early studies reported mainly tau positive intra-neuronal inclusions, but Tar DNA binding protein-43 (TDP-43) positive inclusions and particularly Alzheimer’s disease (AD) pathology have been increasingly recognized. [3], [9]–[12] The ability to accurately detect underlying pathology early in the course of CBS will be crucial when effective therapies are developed. It has been suggested that visuospatial dysfunction on cognitive testing may predict underlying AD, [11] but this has been based upon retrospective case reviews, rather than prospective evaluation using a biomarker of pathology.

The positron emission tomography (PET) ligand [^11^C] Pittsburgh Compound B (PiB) was developed to detect fibrillar β-amyloid peptide, which is a characteristic feature of AD pathology. [13] PiB-PET scanning is a sensitive and specific biomarker of AD pathology, [14], [15] and can be used to detect pathology in vivo in patients with atypical dementia syndromes. The present study aimed to establish clinical, neuropsychological, and neuroimaging features that could be useful in the distinction of CBS due to AD pathology from other CBS cases in life based on PiB status.

## Methods

### Study Population

Patients with CBS were recruited from a specialist cognitive disorders clinic. The diagnosis of CBS was made after detailed clinical assessment by an experienced cognitive neurologist (JRH), neuropsychological evaluation, and diagnostic neuroimaging. All patients met recently proposed criteria for CBS, which require patients to present with an insidiously progressive disorder, which was unresponsive to levodopa treatment. In addition, two of three major features together with two minor features were required for a diagnosis of CBS (See Table 1). [16] Patients with CBS may develop similar symptoms to those seen in frontotemporal dementia. For example, CBS patients may develop progressive speech and language disturbance that resembles the progressive non-fluent aphasia subtype of frontotemporal dementia, or behavioral disturbance – typically apathy rather than disinhibition – similar to patients with behavioral variant frontotemporal dementia. As such, patients with isolated disturbances of speech or behavior, without prominent limb apraxia or akinetic rigid motor symptoms, were excluded from the present study. Patients with other alternative diagnoses notably idiopathic Parkinson’s disease, progressive supranuclear palsy, vascular dementia, or psychiatric disease were also excluded. Age and gender matched control subjects were recruited from a volunteer database. Although control subjects did not undergo PiB-PET imaging, all were screened for evidence of neurological disease and performed normally on neuropsychological testing.

**Table 1:** Proposed criteria for the diagnosis of CBS.

Modified Bak and Hodges criteria (Cambridge criteria)
Mandatory criteria
Insidious onset and gradual progression
No sustained response to levodopa treatment†
Major and minor criteria*
Motor features
*Akinetic rigid syndrome*
Focal or segmental myoclonus
Asymmetrical dystonia
Cortical motor sensory features
*Limb apraxia*
Alien limb phenomenon
Cortical sensory loss or dyscalculia
Cognitive features
*Speech and language impairment* ‡
Frontal executive dysfunction§
Visuospatial deficits

To satisfy the diagnostic criteria, patients had to have an insidiously progressive disorder, which was unresponsive to levodopa treatment, two of three major criteria (in italics), and two minor criteria.

* Criteria in italics are major criteria, and the rest are minor criteria.

† The response of the parkinsonism to levodopa therapy should be tested with at least 25/250 mg of carbidopa/levodopa administered three times a day for at least 2 months. The response to levodopa is considered poor when the extrapyramidal features fail to show marked improvement, or the therapeutic effect is transient (i.e., lasts less than a year).

‡ Includes aphasia, dysarthria and dysgraphia.

§ Includes frontal release signs reduced verbal fluency and other abnormal tests of frontal functions.

Ethics approval for the study was obtained from the Human Research Ethics Committee of the South Eastern Sydney Local Health District – Northern Sector. Participants gave written, informed consent prior to inclusion in the study. In addition, written consent was also obtained by the next-of-kin.

### Clinical Assessments

Detailed clinical assessments were performed on all CBS patients, according to a predefined assessment protocol. A semi-structured questionnaire was used to ensure that all relevant clinical symptoms and signs had been sought and documented during the clinical assessment. In particular, symptoms and signs suggesting disturbances of memory, language, vision or perception, limb function, and motor symptoms such as limb weakness, clumsiness, and alien limb phenomenon were investigated.

Speech and language was assessed for dysarthria, motor speech disorder, phonological errors, agrammatism, word-finding difficulty, anomia, and impaired word and sentence repetition. [17] Apraxia was assessed by imitation of meaningful and meaningless hand gestures in each hand, or meaningful orobuccal gestures, and determined to be either present or absent. In addition, imaginary tool use was assessed and body-part-as-object noted. General neurological examination included an assessment of eye movements, parkinsonian signs such as limb rigidity, tremor, bradykinesia, and gait disturbance, although cortical sensory loss was not consistently examined and was therefore omitted from the present study.

Functional impairment and neuropsychiatric symptoms were assessed using the Cambridge Behavioural Inventory (CBI), [18], [19] a caregiver questionnaire which explores performance on a range of different everyday activities such as memory and orientation, everyday skills, self care, abnormal behavior, mood, beliefs, eating habits, sleep, stereotypic and motor behaviors, and motivation. Since the number of items in each functional domain differs from 2 to 8, the graded CBI responses were converted to a percentage, where a higher percentage indicated a greater level of behavioral disturbance in that particular functional domain.

### Neuropsychological Assessments

General cognitive screening was performed using the Addenbrooke’s Cognitive Examination – Revised (ACE-R), [20] which includes assessments of orientation and memory, language (including object naming, word repetition, reading and writing), verbal fluency (both letter and category), and visuospatial function (including visual interpretation and figure copy). The ACE-R is scored out of a maximum of 100 points, with a score of >88 points indicating normal performance. In addition to total ACE-R scores, sub-scores were calculated including the Attention/Orientation, Memory, Fluency, Language and Visuospatial ACE-R sub-scores. The Folstein Mini-Mental Status Examination (MMSE) was also performed [21].

Visuospatial function was assessed using the Visual Object and Space Perception Battery (VOSP). [22] The VOSP requires the subject to interpret complicated visual scenes; three components were used dot counting, position discrimination, and cube analysis. When patients were capable of holding and manipulating a pencil, the Rey-Osterrieth Complex Figure task was also administered. [23] The Rey-Osterrieth Complex Figure task requires the subject to copy a complicated figure – the accuracy of the copy, approach to the task, and time taken to complete the figure were all noted. Verbal memory was assessed using the Rey Adult Verbal Learning Task (RAVLT), which requires the subject to learn and retain a list of unrelated words. Visual memory was assessed using the Doors and People test, which requires the subject to identify a previously presented door from an array of similar doors. [24] Executive dysfunction was assessed using FAS letter fluency, digit span, and the Trail-Making Test (Parts A and B). FAS letter fluency requires the subject to generate as many words in one minute beginning with the letters “F”, “A”, and “S”. Digit span requires the subject to repeat series of digits both forwards and backwards. The Trail Making Test Part A requires the subject to join circles numbered sequentially (i.e. 1, 2, 3, 4, 5 etc.), while Part B requires the subject to alternate between sequential numbers and letters (i.e. 1, A, 2, B, 3, C etc.). [25] Both the number of errors and the time taken to complete the task were recorded.

### PiB-Positron Emission Tomography

Each CBS patient received ∼370 MBq ^11^C-PiB intravenously over 1 min. A 30-min acquisition in 3D mode starting 40 min after injection of PiB was performed with a Phillips Allegro™ PET camera. A transmission scan was performed for attenuation correction. PET images were reconstructed using a 3D RAMLA algorithm. Images were processed using a preset template of narrow cortical regions of interest placed by an operator (VLV) who was blind to the participant’s clinical status. PET data were not corrected for partial volume effects. Standardized uptake values for PiB were calculated for all brain regions examined, and standardized uptake value ratios were generated by dividing all regional standardized uptake values by the cerebellar cortex standardized uptake values. The cerebellum was chosen as reference, as it is not normally subject to amyloid accumulation. [13] Neocortical β-amyloid burden was expressed as the average standardized uptake value ratios of frontal, superior parietal, lateral temporal, lateral occipital, and anterior and posterior cingulate regions. Given the bimodal distribution of PiB-standardized uptake value ratios that is observed in healthy controls, a hierarchical cluster analysis was performed on all elderly healthy-control participants at Austin Health, n = 118, age 73.2+/−7.4 years, Mini-Mental State Examination 29.2+/−1.0 (mean +/− SD) that yielded a cut-off for ‘high’ or ‘low’ neocortical standardized uptake value ratios of 1.5, consistent with cut-off values used in previous PiB-PET studies. [26], [27] CBS patients with ‘high’ PiB binding (i.e. standardized uptake ratio >1.5) were classified as PiB-positive and those with ‘low’ PiB binding were classified as PiB-negative.

### Voxel-Based Morphometry Analysis

All 14 CBS patients and 20 age-matched healthy controls underwent magnetic resonance imaging according to a standardized protocol using a 3-Tesla Phillips MRI scanner with standard quadrature head coil (8 channels). The 3D T1-weighted sequences were acquired with the following sequences: coronal orientation, matrix 256×256, 200 slices, 1×1 mm2 in-plane resolution, slice thickness 1 mm, TE/TR = 2.6/5.8 ms, and TFE/FFE Pulse sequence. 3D T1-weighted sequences were used to perform a voxel-based morphometry (VBM) analysis using the FSL software package (see http://www.fmrib.ox.ac.uk/fsl/fslvbm/index.html).

Brain extraction was first performed on all scans utilizing the Brain Extraction Tool (BET) algorithm in FSL, after applying a fractional intensity threshold of 0.22. Brain extraction was checked visually for all scans to ensure that no brain matter was excluded. Similarly, the scans were checked to verify that no non-brain matter (e.g. skull, dura mater, optic nerve) were included in the brain extracted images. If brain matter was falsely excluded, or non-brain matter detected, the BET algorithm for that scan was repeated by changing the fractional intensity threshold to give smaller or larger brain outline estimates. A study-specific grey matter template was then created by including 10 scans of each group (total n = 20). The same number of scans across groups was used to avoid any bias during the registration step (i.e. favoring one group) while at the same time representing all included groups equally. The template scans were then registered to the Montreal Neurological Institute Standard space (MNI 152) using non-linear b-spline representation of the registration warp field resulting in study-specific grey matter template at 2×2×2 mm^3^ resolution in standard space. At the same time, all brain extracted scans were also processed with the FMRIB’s Automatic Segmentation Tool (FAST v 4.0) to achieve tissue segmentation of i) grey matter, ii) white matter and iii) CSF via a hidden Markov random field model and an associated Expectation-Maximization algorithm. The FAST algorithm also corrected for spatial intensity variations such as bias field or radio-frequency inhomogeneities in the scans, resulting in partial volume maps of the scans. Subsequently, the grey matter partial volume maps were registered to the study-specific template via non-linear b-spline representation of the registration warp and modulated by dividing them by the Jacobian of the warp field, to correct for the contraction/enlargement due to the non-linear component of the transformation. Finally, the normalized and modulated grey matter maps were smoothed with an isotropic Gaussian kernel (standard deviation = 3 mm; full width half maximum = 8 mm). The statistical analysis was performed by employing a voxel-wise general linear model. Significant clusters were formed by employing the threshold-free cluster enhancement (TFCE) method. The TFCE method is a cluster-based thresholding method which does not require the setting of an arbitrary cluster forming threshold (e.g. t, z <4); instead, it takes a raw statistics image and produces an output image in which the voxel-wise values represent the amount of cluster-like local spatial support. The TFCE image was then converted into voxel-wise p-values via permutation testing. Permutation-based non-parametric testing with 5000 permutations was performed. All group comparisons were tested for significance at p<0.05, corrected for multiple comparisons via Family-wise Error correction across space. Age and ACE-R were entered as covariates in the model, but TIV and gender were not as the Jacobian modulation step did not include the affine part of the registration. As such, the data was already normalized for head size as a scaling effect. As the data was scaled for head size, inclusion of gender as a covariate was unnecessary.

### Statistical Analysis

Statistical analysis was performed by a single author (JRB) using the Statistical Package for Social Sciences (version 19.0, SPSS Inc.; Chicago, IL, USA). Comparisons were first made between patients with CBS and control subjects. CBS patients were then grouped according to PiB status, as either PiB-positive or PiB-negative, and these groups were compared to each other and to controls. Continuous variables were analyzed using analysis of variance (ANOVA) when normally distributed or the Kruskal–Wallis test when non-normally distributed. Pair-wise comparisons were performed using the independent samples t test when continuous variables were normally distributed and the Mann-Whitney test when non-normally distributed. Categorical data were analyzed using the Chi-Square test.

## Results

### Patient Demographics and Clinical Features

In total, 14 CBS patients and 20 control subjects were included. The mean age of CBS patients was 66.1+/−6.9 years, male and female gender was split equally, and the median symptom duration was 35.5+/−22.6 months. The mean duration of formal education did not differ between the two groups (CBS 11.4 years, controls 13.1 years, NS). Of the 14 CBS patients four (28.6%) were PiB-positive and the remaining 10 (71.4%) were PiB-negative. The pattern of β-amyloid distribution in PiB-positive patients did not differ from that seen in previous cohorts of AD patients.

Of the 14 patients, 10 (71.4%) presented with language dysfunction as the initial symptom. The most common language abnormalities were impaired single word repetition (61.5%), dysgraphia (58.3%), phonological errors in spontaneous speech (46.2%), impaired sentence repetition (38.5%), and word-finding difficulty (30.8%). Agrammatism and anomia were only occasionally identified. Significant impairment was detected in multiple functional domains on the CBI (Table 2).

**Table 2:** Neuropsychological performance of CBS patients compared to controls.

	CBS	Control	P-Value
**ACE-R and MMSE**			
Attention	17 (5–18)	18 (16–18)	NS
Memory	21.5 (3–26)	25 (21–26)	<0.05
Fluency	9.5 (0–12)	13 (8–14)	<0.05
Language	22 (5–26)	26 (23–26)	<0.001
Visuospatial	12.5 (2–16)	16 (14–16)	<0.001
ACE-R Total	81.5 (24–91)	95 (88–100)	<0.05
MMSE	26 (7–29)	29 (27–30)	<0.001
**BEHAVIOUR – CBI**			
Memory and Orientation (%)	32.8 (0–65.6)	3.1 (0–37.5)	<0.05
Everyday Skills (%)	37.5 (0–80)	0 (0–10)	<0.001
Self Care (%)	18.8 (0–81.3)	0 (0–0)	<0.001
Abnormal Behaviour (%)	8.3 (0–29.2)	0 (0–12.5)	<0.05
Mood (% subscore)	18.8 (0–56.3)	0 (0–18.8)	<0.001
Beliefs (% subscore)	0 (0–41.7)	0 (0–0)	NS
Eating Habits (% subscore)	6.3 (0–31.3)	0 (0–25)	NS
Sleep (% subscore)	37.5 (0–100)	0 (0–50)	<0.05
Stereotypic and Motor Behaviours (% subscore)	9.4 (0–31.3)	0 (0–50)	NS
Motivation (% subscore)	27.5 (0–80)	0 (0–15)	<0.001
Total (%)	22.8 (7.8–40)	2.8 (0–13.3)	<0.001
**VISUOPATIAL FUNCTION**			
**- Visual Object and Space Perception Battery (VOSP)**			
Dots (# identified)	9.5 (4–10)	10 (8–10)	NS
Position	19.5 (16–20)	20 (19–20)	NS
Cube	8 (0–10)	10 (7–10)	NS
**- Rey-Osterrieth Complex Figure**			
Copy Score	25.5 (3–33)	34 (25.5–36)	<0.001
Recall Score (at 3 minutes)	12.5 (1–19.5)	16.5 (8–35)	<0.05
**MEMORY**			
**Verbal Memory – Rey Adult Verbal Learning Task (RAVLT)**			
Immediate recall	7 (0–12)	10.5 (4–15)	<0.05
Recall at 30 Mins	6.5 (0–11)	10.5 (4–15)	<0.001
**Visual Memory – The Doors and People test**			
Combined (Raw score)	12.5 (0–19)	19 (0–24)	<0.001
**EXECUTIVE FUNCTION**			
**– FAS Letter Fluency**			
Number of Correct Responses	10.5 (0–17)	15 (6–23)	<0.001
**– Trail Making Test**			
Part A time (seconds)	79 (23–320)	28 (21–72)	<0.05
Part B time (seconds)	172 (74–273)	72 (42–163)	<0.05
**– Digit Span**			
Forwards (Raw score)	7 (3–14)	11.5 (8–14)	<0.05
Backwards (Raw score)	4 (0–9)	7 (1–13)	<0.001

Patients with CBS were at least moderately impaired on neuropsychological testing. The ACE-R total and MMSE were significantly reduced in CBS patients compared to controls. Apart from Attention, all other ACE-R sub-scores were significantly reduced with the greatest decline from normal values seen in the Fluency and Visuospatial sub-scores. Formal neuropsychological evaluation confirmed impairment of memory, as well as visuospatial and executive impairment. Note: some tasks that required manipulation of a pencil, such as the Rey-Osterrieth Complex Figure and Trails, had to be abandoned in some CBS patients due to severe apraxia of the dominant limb. All data represented as median (minimum - maximum).

The motor examination in CBS patients was characterized by marked limb apraxia and parkinsonism. All had difficulty performing meaningless hand gestures, while 91.7% had difficulty producing meaningful hand gestures and 69.2% had evidence of orobuccal apraxia. Tool usage was impaired in 63.6%. Limb rigidity was detected in 92.3%, and all patients demonstrated limb bradykinesia. Other motor signs were much less common, for example myoclonus and alien limb phenomenon were each present in 22.2%, and dystonia was detected in only 11.1% of CBS patients.

### Clinical Features According to PiB Status

There was no significant difference in median age between PiB-positive (60 years; range 57–74 years) and PiB-negative cases (69 years; range 59–80 years). In addition, the mean symptom duration and duration of formal education did not differ between PiB-positive and PiB-negative groups (Table 3). Overall, there were no significant differences in motor examination findings between the two CBS groups (Table 4). Interestingly, alien limb phenomenon was detected in all PiB-positive cases but only 12.5% of PiB-negative cases (P = 0.11). Furthermore, some degree of orobuccal apraxia was found in 55.6% of PiB-negative cases, but none of the PiB-positive cases (P = 0.11).

**Table 3:** Demographic and functional characteristics of CBS patients according to PiB status.

	PiB-positive	PiB-negative	Control	P-Value
Number	**4**	**10**	**20**	
Age (years +/− SD)	62.8+/−7.7	67.4+/−6.6	66.9+/−5.4	NS
Male (% subjects)	1(25%)	6 (60%)	10 (50%)	NS
Education (years +/− SD)	11.3+/−3.9	11.5+/−3.0	13.1+/−2.4	NS
Symptom Duration (months +/− SD)				
Mean	57.8+/−19.2	39.5+/−22.5	N/A	NS
**ACE-R and MMSE**				
Attention	11 (5–18)	18 (14–18)	18 (16–18)	NS
Memory	12 (3–20)	23 (20–26)	25 (21–26)	<0.001a,b,c
Fluency	6 (1–12)	9.5 (0–12)	13 (8–14)	<0.05b,c
Language	16 (5–24)	22.5 (17–26)	26 (23–26)	<0.001b,c
Visuospatial	6.5 (2–12)	14 (8–16)	16 (14–16)	<0.001a,b,c
Total	47.5 (24–86)	84 (72–91)	95 (88–100)	<0.001b,c
MMSE	16.5 (7–27)	26 (22–29)	29 (27–30)	<0.001b,c
**BEHAVIOUR – CBI**				
Memory and Orientation (% subscore)	56.3 (43.8–65.6)	14.1 (0–59.4)	3.1 (0–37.5)	<0.05a,c
Everyday Skills (% subscore)	67.5 (35–80)	32.5 (0–50)	0 (0–10)	<0.001a,b,c
Self Care (% subscore)	37.5 (12.5–68.8)	15.6 (0–81.3)	0 (0–0)	<0.001b,c
Abnormal (% subscore)	6.3 (0–29.2)	8.3 (0–20.8)	0 (0–12.5)	<0.05^b^
Mood (% subscore)	25 (0–56.3)	18.8 (0–50)	0 (0–18.8)	<0.001b,c
Beliefs (% subscore)	0 (0–0)	0 (0–41.7)	0 (0–0)	NS
Eating Habits (% subscore)	21.9 (12.5–31.3)	0 (0–25)	0 (0–25)	<0.05^c^
Sleep (% subscore)	43.8 (0–62.5)	31.3 (0–100)	0 (0–50)	<0.05^b^
Stereotypic and Motor Behaviours (% subscore)	9.4 (0–25)	9.4 (0–31.3)	0 (0–50)	NS
Motivation (% subscore)	32.5 (10–45)	27.5 (0–80)	0 (0–15)	<0.001b,c
Total (%)	31.4 (25–40)	16.9 (7.8–37.8)	5 (0–24)	<0.001b,c

There were no significant differences between PiB-positive and PiB-negative CBS patients, or controls in age, gender, mean education or symptom duration. Although PiB-negative CBS patients were cognitively and functionally impaired compared to controls, PiB-positive cases demonstrated greater impairment overall. All neuropsychological and behavioral data represented as median (minimum - maximum).

a PiB-positive v PiB-negative, P<0.05.

b PiB-negative v Controls, P<0.05.

c PiB-positive v Controls, P<0.05.

**Table 4:** Motor features of CBS patients according to PiB status.

	PiB-positive	PiB-negative	P - Value
**APRAXIA**			
**- Orobuccal Apraxia**	0	4 (44.4%)	0.11
**- Impaired meaningless hand gestures**	4 (100%)	7 (87.5%)	NS
**- Impaired meaningful hand gestures**	4 (100%)	9 (100%)	N/A
**- Impaired tool use**	2 (50%)	5 (71.4%)	NS
**ALIEN LIMB PHENOMENON**	4 (100%)	1 (12.5%)	0.11
**PARKINSONIAN SIGNS**			
**- Rigidity**	3 (75%)	9 (100%)	NS
**- Bradykinesia**	4 (100%)	9 (100%)	N/A
**- Tremor**	0	1 (11.1%)	NS

There were no significant differences in the motor features between PiB-positive and PiB-negative patient groups, although there was a trend for increased alien limb phenomenon in PiB-positive cases and for increased orobuccal apraxia in PiB-negative cases. Note detailed information on motor features was missing in one PiB-negative case.

Language impairment was present in both the PiB-positive and PiB-negative CBS groups. There was a trend for greater impairment of sentence repetition in PiB-positive cases (PiB-positive 75%; PiB-negative 22.2%, P = 0.07). In addition, dysgraphia was present in all PiB-positive and only 44.4% of PiB-negative cases (P = 0.09).

**Figure 1:**
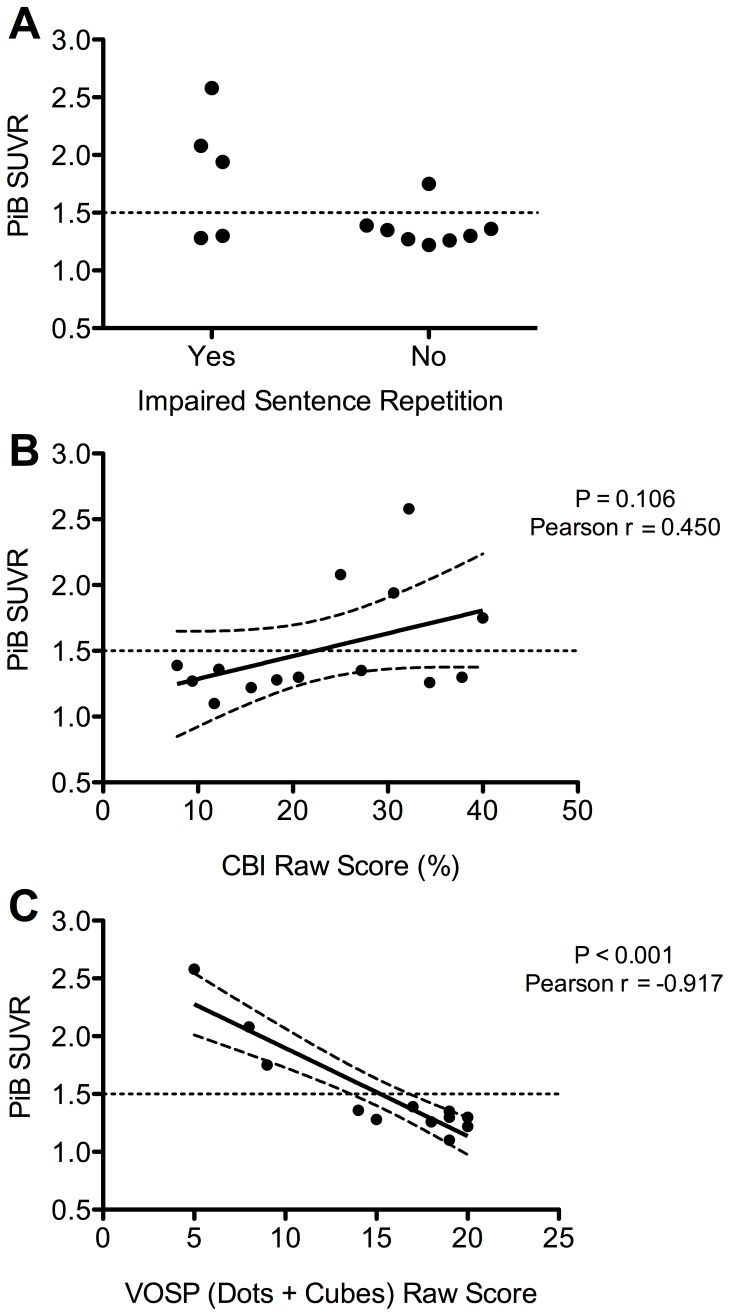
Correlates of PiB-binding in CBS patients. A – Impaired sentence repetition was detected in 75% of PiB-positive patients (defined as an SUVR ratio of >1.5) compared to only 22.2% of PiB-negative patients (P = 0.07). **B** – There was a trend (P = 1.06) for a correlation between increased PiB-binding (reflected in increased SUVR ratio) and functional impairment (reflected by an increased CBI total). **C** – Increased PiB-binding (reflected by an increased SUVR) was strongly and highly significantly (P<0.001) correlated with visuospatial dysfunction. Note – PiB = Pittsburgh Compound B, SUVR = standardized uptake value ratio, CBI = Cambridge Behavioural Inventory, VOSP = Visual Object and Space Perception Battery. In Figure 1B and 1C the line of best fit is shown, with 95% confidence bands.

Functional impairment was common in both groups (Table 3), but more severe in PiB-positive CBS patients. Specifically, when the PiB-positive, PiB-negative and control groups were compared, there was a significant inter-group difference in the CBI total, with post-hoc analyses demonstrating a significantly (P<0.05) increased CBI total in PiB-positive patients (31.9% +/−6.2%) compared to PiB-negative (19.5% +/−10.5%) patients and controls (3.8% +/−3.9%). Consistent with this finding, there was a trend (P = 0.106) for a correlation (Pearson r = 0.450) between PiB binding (reflected in an increased standardized uptake value ratio) and an increased CBI total score (Figure 1B). PiB-positive patients were particularly impaired in the memory and everyday skills domains.

### Neuropsychological Performance

Patients with CBS were at least moderately impaired compared to controls on neuropsychological testing, as reported in Table 2. The ACE-R total and MMSE were significantly reduced in CBS patients compared to controls. Apart from Attention, all other ACE-R sub-scores were significantly reduced with the greatest decline from normal values seen in the Fluency and Visuospatial sub-scores.

Formal neuropsychological evaluation confirmed visuospatial, executive, verbal memory, and visual memory impairment (Table 2). Specifically, CBS patients were impaired on the Rey Complex Figure copy (P<0.001) and recall (P<0.05). Verbal memory was impaired as reflected in reduced immediate (P<0.05) and delayed (P<0.001) recall on the RAVLT, as was visual memory as indicated by poor performance on the Doors and People test (P<0.001). Executive dysfunction was evident from a reduced number of correct responses on FAS letter fluency (P<0.001), prolonged Trail-Making test times, and reduced digit span – both forwards (P<0.05) and backwards (P<0.001).

### Neuropsychological Performance According to PiB Status

PiB-positive CBS patients demonstrated greater cognitive impairment than PiB-negative patients (Table 3). Compared to controls, both CBS groups performed significantly (P<0.05) worse on all components of the ACE-R (apart from the Attention subtask), and on the total score, but PiB-positive patients performed significantly (P<0.05) worse on the visuospatial sub-score compared to PiB-negative patients. PiB-positive CBS patients demonstrated greater visuospatial dysfunction, with significantly (P<0.05) worse performance on the “Dots” and “Cubes” sections of the VOSP (Table 5). Consistent with this finding, there was a very strong (Pearson r = -0.917) and highly significant (P<0.001) correlation between PiB binding (reflected by an increased standardized uptake value ratio) and impaired performance on the “Dots” and “Cubes” sections of the VOSP (Figure 1C). PiB-positive CBS patients demonstrated greater executive dysfunction with a significant reduction in performance on digit span forwards (P<0.05, Table 5). There were trends for prolonged Trail Making Test Part A time (P = 0.06), reduced immediate (P = 0.07) and delayed (P = 0.09) recall on the RAVLT verbal memory task, but no significant differences in visual memory, as assessed by the Doors and People test.

**Table 5:** Neuropsychological profile of CBS patients according to PiB status.

	PiB-positive	PiB-negative	Controls	P-Value
**VISUOPATIAL FUNCTION**				
**- Visual Object and Space Perception Battery (VOSP)**				
Dots (# identified)	5 (4−9)	10 (7−10)	10 (8–10)	<0.05^a^
Position	18 (16–18)	20 (16–20)	20 (19–20)	<0.05
Cube	0 (0–4)	9 (4–10)	10 (7–10)	<0.05a,c
**- Rey-Osterrieth Complex Figure**				
Copy Score	3 (3–3)	26 (20.5–33)	34 (25.5–36)	<0.05b,c
Recall Score (at 3 minutes)	N/A	12.5 (1–19.5)	16.5 (8–35)	<0.05b,c
**MEMORY**				
**Verbal Memory – Rey Adult Verbal Learning Task (RAVLT)**				
Immediate recall	2 (0–7)	8 (0–12)	10.5 (4–15)	<0.05^c^
Recall at 30 Mins	1 (0–6)	7 (0–11)	10.5 (4–15)	<0.05b,c
**Visual Memory – The Doors and People test**				
Combined (Raw score)	6 (0–16)	13.5 (0–19)	19 (0–24)	<0.05b,c
**EXECUTIVE FUNCTION**				
**– FAS Letter Fluency**				
Number of Correct Responses	7.5 (2–12)	10.5 (0–17)	15 (6–23)	<0.05b,c
**– Trail Making Test**				
Part A time (seconds)	233 (146–320)	69 (23–158)	28 (21–72)	<0.001b,c
Part B time (seconds)	243 (243–243)	146 (74–273)	72 (42–163)	<0.05b,c
**– Digit Span**				
Forwards (Raw score)	5 (3–8)	8 (6–14)	11.5 (8–14)	<0.05a,c
Backwards (Raw score)	3 (0–5)	4.5 (3–9)	7 (1–13)	<0.05b,c

a PiB-positive v PiB-negative, P<0.05.

b PiB-negative v Controls, P<0.05.

c PiB-positive v Controls, P<0.05.

Although both CBS groups demonstrated cognitive impairment compared to controls, PiB-positive CBS patients demonstrated more cognitive impairment than PiB-negative cases, with significant visuospatial impairment, and executive dysfunction. In addition, there was a trend for impaired memory in the PiB-positive group. Note: some tasks that required manipulation of a pencil, such as the Rey-Osterrieth Complex Figure and Trails, had to be abandoned in some CBS patients due to severe apraxia of the dominant limb.

### Voxel-based Morphometry Analysis

**Figure 2:**
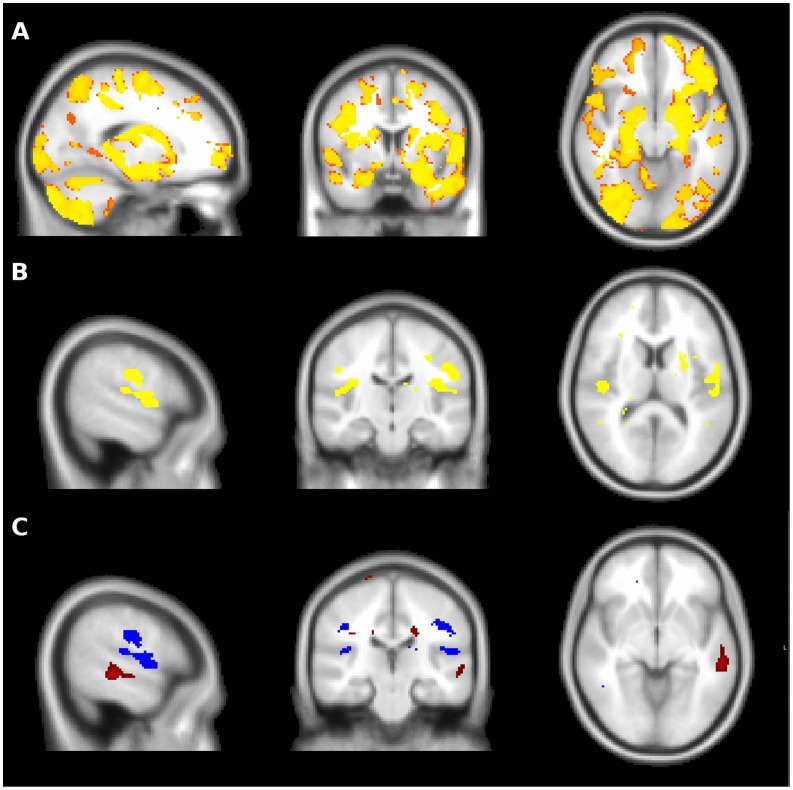
Voxel based morphometry in CBS patients. **A –** CBS patients demonstrated widespread cerebral atrophy compared to controls, with marked frontal, temporal, parietal and basal ganglia involvement. **B** – The PiB-positive and PiB-negative groups both demonstrated bilateral peri-insular and post-central gyrus atrophy, worse on the left than the right. **C** – In addition to peri-insular atrophy (blue), the PiB-positive group demonstrated atrophy of the posterior portion of the left superior temporal gyrus when compared to the PiB-negative group (red). In the inverse contrast, the PiB-negative group did not demonstrate greater atrophy than the PiB-positive group in any brain region.

Separate voxel-based morphometry analyses were performed, initially comparing all CBS patients to controls. As demonstrated in Figure 2A, CBS patients had widespread cerebral atrophy with marked frontal, temporal, parietal, and basal ganglia involvement. A conjunction analysis of CBS patients classified according to PiB status was then conducted to reveal atrophy common to both groups. Significant bilateral peri-insular atrophy was noted in both groups, slightly more prominent on the left than the right hand side (Figure 2B). In addition, both groups demonstrated atrophy of the post-central gyrus, again more prominent on the left than the right hand side. The PiB-positive group also demonstrated atrophy of the posterior portion of the left superior temporal gyrus (Figure 2C).

## Discussion

Our study, the first to use β-amyloid imaging in patients with CBS, found that of 14 patients reaching strict criteria for CBS four were PiB-positive indicating underlying AD pathology. Subtle differences in the clinical presentation were noted between groups, with greater impairment of visuospatial function, more frequent deficits in sentence repetition, and greater functional decline in PiB-positive cases. Although VBM analyses confirmed considerable overlap between CBS subgroups, atrophy affecting the posterior part of the left superior temporal gyrus distinguished PiB-positive cases. Taken together, these clinical, neuropsychological, and imaging characteristics may be useful in detecting underlying AD pathology in CBS.

Before considering the findings of the present study in greater detail, it is acknowledged that patients were recruited from a cognitive disorders clinic, rather than a movement disorders clinic, and this may introduced an element of referral as well as ascertainment bias. For example, the Unified Parkinson’s Disease Rating Scale (UPDRS) was not consistently applied to the assessment of CBS patients in the present study. Furthermore, the measures used to document functional impairment in the present study, while sensitive to cognitive and behavioral disturbance, may be insensitive to motor functional impairment. Reflecting the rarity of the disease, the number of participants in the present study was small which limited statistical power in comparisons of clinical, neuropsychological, and neuroimaging features of PiB-positive and PiB-negative patients, particularly as PiB-positive patients exhibited greater global cognitive impairment. In addition, since PiB imaging only detects the presence of amyloid deposition, the clinical and neuropsychological characteristics of CBS due to other specific pathologies could not be addressed by the present study. Furthermore, it is acknowledged that the presence of underlying Alzheimer’s pathology, as detected by PiB imaging, does not exclude the possibility of a concomitant neuropathological diagnosis as the cause of CBS. Finally, longer duration of symptoms or more severe pathology could potentially explain differences in cognitive performance observed in the PiB-positive group, rather than specifically reflecting underlying AD pathology.

The finding of 28.6% (4/14) positivity on PiB imaging is consistent with the growing evidence for pathological heterogeneity in CBS and that a significant proportion of patients have AD as the underlying pathology. Estimates from postmortem studies have indicated a rate of AD pathology between 23 and 50%. [3], [10], [11], [28] The patients with AD in our series were largely indistinguishable from PiB-negative cases at a clinical level, had equivalent severity of apraxia, and fulfilled the newly proposed criteria for CBS. [16] Interestingly, all showed alien limb phenomenon – often regarded as a classic hallmark of corticobasal degeneration. A previous study demonstrated a trend towards increased alien limb phenomenon in CBS due to AD, as well as a significant increase in the frequency of myoclonus. [28] In addition, only PiB-negative patients demonstrated orobuccal apraxia, possibly reflecting the predilection for pathology in PiB-positive cases to be located more posteriorly, rather than in the peri-insular region. While preliminary, these observations suggest that differences in motor symptoms and signs may prove useful in identifying AD pathology in CBS.

Aphasia is a common feature in CBS. [11], [28] The range of language abnormalities reported to date may reflect the pathologic heterogeneity of the syndrome. Our findings suggest that impaired sentence repetition, in contrast to speech apraxia, may distinguish AD from tauopathy cases, although the language profiles of CBS due to other pathologies such as PSP and TDP-43 remain to be elucidated. Impaired sentence repetition is of particular interest in the setting of logopenic progressive aphasia (LPA) which is now recognized as the third variant of primary progressive aphasia. [29], [30] The underlying pathological substrate of LPA is typically AD, as evident from the postmortem of a small number of cases [31] and β-amyloid PiB imaging in vivo. [17], [32] Thus it appears that LPA and CBS secondary to AD pathology overlap in that both are characterized by disproportionate disturbance of sentence and phrase repetition perhaps due to reduced verbal short-term memory [17], [29], [33].

As would be predicted given the range of underlying pathologies, the pattern of cerebral atrophy reported in CBS has been highly variable, but typically involves the prefrontal, temporal, and parietal lobes as well as the basal ganglia. [10], [11], [34], [35] The present study found that peri-insular atrophy was common to both PiB-positive and PiB-negative groups, but PiB-positive cases had significant atrophy involving the posterior portion of the left superior temporal gyrus. This finding is consistent with recent studies that have linked atrophy of the posterior temporal lobe to AD pathology in various settings. Atrophy of the posterior temporal lobe was described in early reports of LPA, [29] and sentence repetition, a key clinical feature of LPA, has been linked to atrophy in superior temporal gyrus. [36] Furthermore, recent postmortem studies have suggested that posterior temporal and inferior parietal atrophy may be predictive of AD pathology in CBS, [10], [35] and some have even suggested that atrophy of the posterior temporal lobe is predictive of AD pathology regardless of the clinical phenotype. [37] In this context, the findings of the present study suggest that atrophy of posterior portion of the left superior temporal gyrus may prove useful in identifying individual CBS cases due to AD pathology.

Patients with PiB-positive CBS had more significant functional and cognitive impairment than PiB-negative patients, which may be another clue to underlying AD pathology. As assessed by the CBI, PiB-positive patients had more functional impairment and the pattern of disturbance reflected the severity and pattern of cognitive deficits. For example, carers of PiB-positive patients reported impaired memory and poor everyday skills. Furthermore, there was a trend for increased PiB binding to be correlated with increased behavioral disturbance as measured by the CBI, which is particularly sensitive to cognitive and behavioral disturbances, rather than motor disability. Consistent with this finding, cognitive dysfunction was more severe in PiB-positive patients, with particular impairment on visuospatial tasks. In fact, visuospatial dysfunction was tightly correlated with PiB binding, suggesting that visuospatial impairment may prove a useful marker of Alzheimer’s pathology in CBS. Although many of the visuospatial tasks used in the present study require the subject to draw and may therefore have been confounded by limb apraxia, PiB-positive patients performed worse on the VOSP, which does not require manual dexterity to be completed. The role of neuropsychology in predicting pathology in CBS remains uncertain. One previous study reported no difference in cognitive deficits between CBS due to AD and CBS due to tau pathology, but performance on visuospatial tasks was not reported. [28] In contrast, and consistent with our findings, a more recent study found visuospatial dysfunction to be a key identifier of CBS due to AD pathology [11].

Our study has demonstrated that further characterization of the clinical, imaging and neuropsychological features of CBS patients may be helpful in identifying AD pathology. Our classification of groups requires pathological confirmation, but PiB-PET imaging is sensitive and specific for AD pathology, and may allow detection of pathology at an early stage of disease. Nonetheless, a detailed understanding of clinical, neuropsychological and structural features of CBS due to different pathologies is needed as PiB-PET scanning is not widely available and biomarkers of other pathologies in CBS have not yet been developed.
